# Hypoxia-regulated secretion of IL-12 enhances antitumor activity and safety of CD19 CAR-T cells in the treatment of DLBCL

**DOI:** 10.1016/j.omto.2023.08.009

**Published:** 2023-08-18

**Authors:** Wenping Zhou, Jinxin Miao, Zhenguo Cheng, Zhimin Wang, Jianyao Wang, Haoran Guo, Pengju Wang, Shuangshuang Lu, Lingling Si, Zhongxian Zhang, Louisa Chard Dunmall, Yanyan Liu, Nicholas R. Lemoine, Yaohe Wang

**Affiliations:** 1 Sino-British Research Centre for Molecular Oncology, National Centre for International Research in Cell and Gene Therapy, School of Basic Medical Sciences, Academy of Medical Sciences, Zhengzhou University, Zhengzhou 450052, China; 2 Department of Internal Medicine, The Affiliated Cancer Hospital of Zhengzhou University & Henan Cancer Hospital, Zhengzhou 450008, China; 3 Academy of Chinese Medical Sciences, Henan University of Chinese Medicine, Zhengzhou 45006, China; 4 Center for Cancer Biomarkers & Biotherapeutics, Barts Cancer Institute, Queen Mary University of London, EC1M 6BQ London, UK

**Keywords:** CAR-T cells, DLBCL, ODD, IL-12, Syrian hamster model

## Abstract

CD19-targeted chimeric antigen receptor-modified T (CD19 CAR-T) cell therapy has been demonstrated as one of the most promising therapeutic strategies for treating B cell malignancies. However, it has shown limited treatment efficacy for diffuse large B cell lymphoma (DLBCL). This is, in part, due to the tumor heterogeneity and the hostile tumor microenvironment. Human interleukin-12 (IL-12), as a potent antitumor cytokine, has delivered encouraging outcomes in preclinical studies of DLBCL. However, potentially lethal toxicity associated with systemic administration precludes its clinical application. Here, an armed CD19 CAR expressing hypoxia-regulated IL-12 was developed (CAR19/hIL12ODD). In this vector, IL-12 secretion was restricted to hypoxic microenvironments within the tumor site by fusion of IL-12 with the oxygen degradation domain (ODD) of HIF1α. *In vitro*, CAR19/hIL12ODD-T cells could only secrete bioactive IL-12 under hypoxic conditions, accompanied by enhanced proliferation, robust IFN-γ secretion, increased abundance of CD4+, and central memory T cell phenotype. *In vivo*, adoptive transfer of CAR19/hIL12ODD-T cells significantly enhanced regression of large, established DLBCL xenografts in a novel immunodeficient Syrian hamster model. Notably, this targeted and controlled IL-12 treatment was without toxicity in this model. Taken together, our results suggest that armed CD19 CARs with hypoxia-controlled IL-12 (CAR19/hIL12ODD) might be a promising and safer approach for treating DLBCL.

## Graphical abstract

**Figure:**
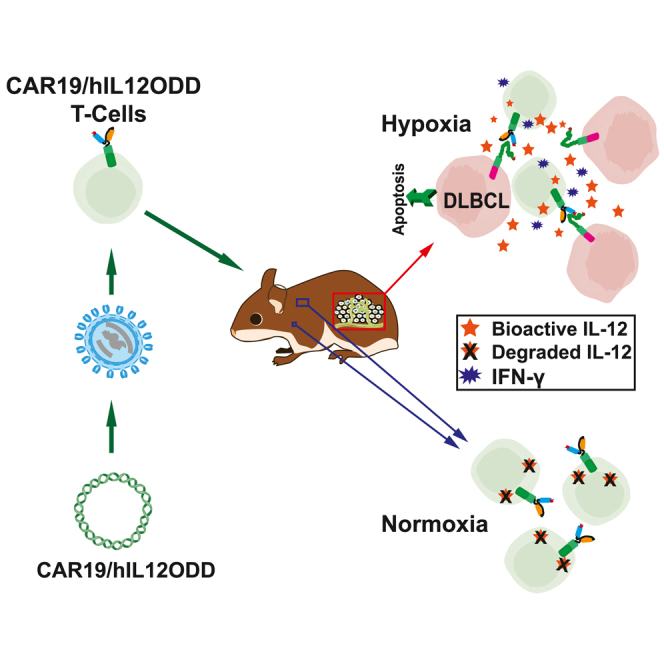

## Teaser

A CD19 CAR expressing hypoxia-regulated IL-12 was developed (CAR19/hIL12ODD), in which IL-12 expression is regulated by the hypoxia through fusion ODD domain of HIF1α. This novel CAR restricts the expression of IL-12 within tumors presenting hypoxic microenvironment and could boost the antitumor activity of CD19 CAR-T cells without systemic toxicity.

## Introduction

Diffuse large B cell lymphoma (DLBCL) is the most common lymphoma,^1^ accounting for approximately 30% of non-Hodgkin lymphoma.^2^ Despite the advanced stage at presentation in the majority of patients, more than 70% can be cured with multimodality therapeutic approaches that include R-CHOP (rituximab, cyclophosphamide, doxorubicin, vincristine, and prednisone) immunochemotherapy,^3^ involved field radiation therapy (RT)^4^ and autologous or allogeneic stem cell transplantation.^3^ However, approximately 30% of patients with multimodality treatment failure have an unacceptably poor prognosis.^5^^,^^6^ This highlights the need to develop an effective, life-prolonging therapeutic option for DLBCL patients with multimodality treatment failure or refractory or relapsed tumors.

Chimeric antigen receptor-modified T (CAR-T) cell therapy has been demonstrated as a promising treatment for malignant diseases in recent years.^7^^,^^8^ In clinical studies, CD19 CAR-T cells have exhibited impressive efficacy with a complete response (CR) rate of ≥90% against B cell acute lymphoblastic leukemia (B-ALL)^9^ and chronic lymphocytic leukemia.^10^ However, in DLBCL, only a 50% CR rate has been achieved and for relapsed/refractory patients ≤30%.^11^ The poor T cell infiltration, persistence, and proliferation following adoptive transfer due to the tumor immunosuppressive microenvironment present as a major impediment that limits the efficacy of CD19 CAR-T cells in treating DLBCL.^12^^,^^13^ Genetic modifications to CAR constructs with immuno-stimulatory cytokines or ligands have been reported to improve the efficacy of CAR-T cells by overcoming the suppressive microenvironment.^14^^,^^15^^,^^16^^,^^17^

Human interleukin-12 (IL-12) has emerged as one of the most potent agents for antitumor immunotherapy due to its multiple functions on immune cells, in particular on T cells,^18^^,^^19^^,^^20^ and to reprogram myeloid-derived suppressor cells.^21^ In accordance with its antitumor activity, IL-12-based clinical trials have demonstrated sustained therapeutic efficacy in multiple cancers.^22^^,^^23^ Notably, for DLBCL, application of IL-12 as monotherapy or combined with rituximab showed encouraging outcomes with objective responses in 60% of patients. Unfortunately, the occurrence of severe side effects associated with lethal inflammatory syndrome poses a challenge for the clinical application of IL-12-based therapy.^23^^,^^24^ To date, IL-12-armed CAR-T cells have shown enhanced antitumor efficacy against a diverse range of murine tumor models of melanoma,^25^ ovarian tumors,^15^ and hepatocellular carcinoma^26^ in preclinical studies. However, IL-12-armed CAR-T therapy has still shown toxic side effects attributable to the systemic secretion of IL-12 as seen in clinical trials.^27^ The efficacy of IL-12 has prompted us to design a new strategy for its delivery in combination with CD19-based CAR-T therapy. We have developed IL-12-armed CAR-T cells expressing modified IL-12 to increase its safety and efficacy. Hypoxia is a feature common to many tumor types, and hypoxic regions are often treatment refractory. Given its ubiquity, using hypoxia to regulate treatment delivery represents a viable option for CAR-T therapy targeting strategies. DLBCL usually occurs in lymph nodes, where hypoxic conditions^28^ and high HIF1α expression are found.^29^ CAR19/hIL12ODD was developed, in which IL-12 expression is regulated by the hypoxic tumor microenvironment through fusion with the ODD domain of HIF1a.^30^ This modification restricts the expression of IL-12 to within tumors, where a hypoxic microenvironment is present. Human IL-12 is non-functional in murine models, but we have demonstrated that it is active in Syrian hamster systems.^31^ We used a recently reported immunodeficient Syrian hamster model (named ZZU001)^32^ to assess the antitumor efficacy and safety of CAR19/hIL12ODD. Our *in vitro* and *in vivo* data demonstrated that CAR19/hIL12ODD has potent antitumor activity and was safe for the treatment of DLBCL.

## Results

### Design and construction of a hypoxia-dependent IL-12-secreting CD19 CAR

**Figure 1:**
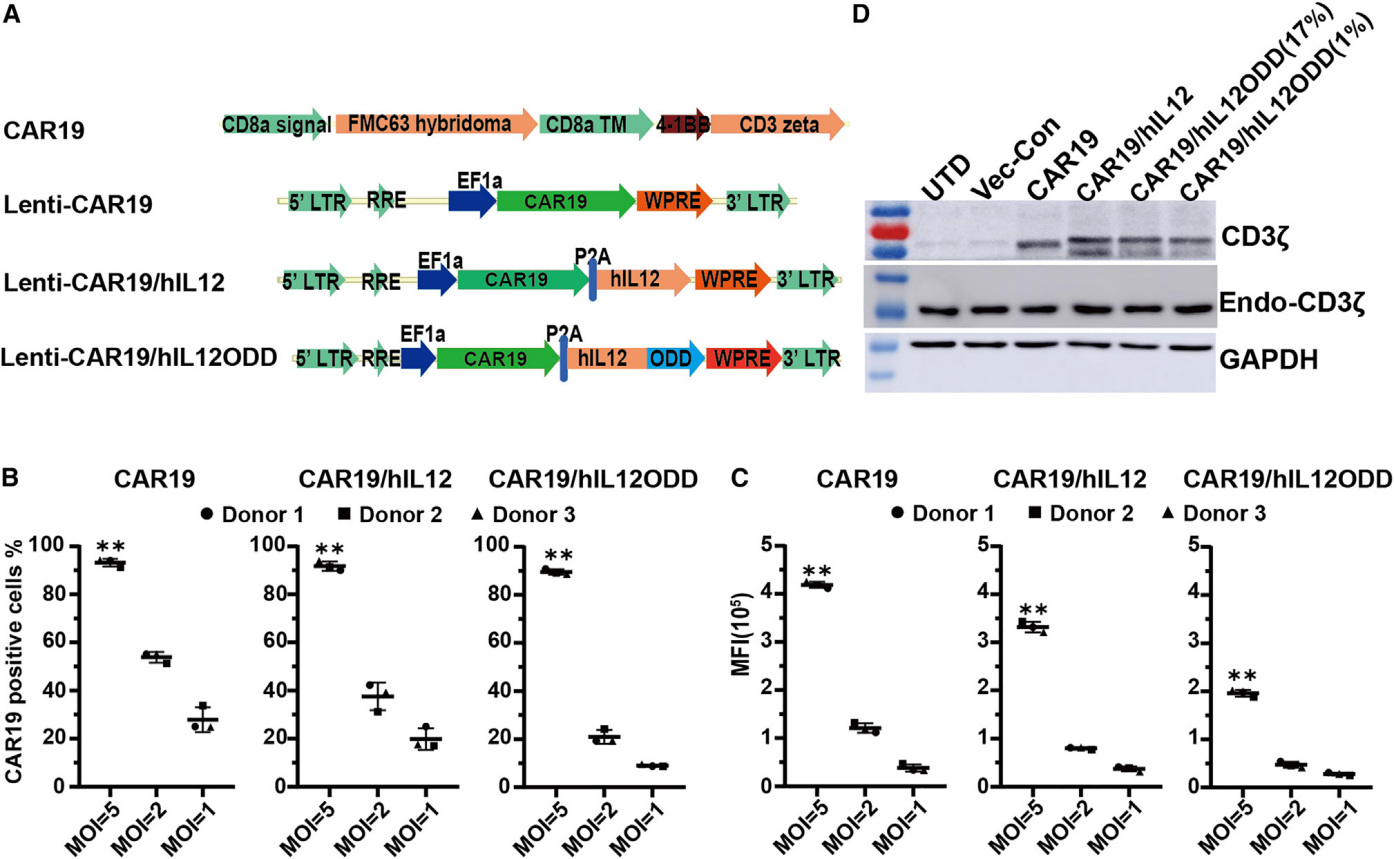
Construction of hypoxia-dependent IL-12 secreting CD19 CAR-T cells (A) Schematic representation of human CD19-specific CAR (CAR19), IL-12-armed CAR19 (CAR19/hIL12), and hypoxia-dependent IL-12 secreting CAR19 (CAR19/hIL12ODD). Human CD3 T^+^ cells were isolated and activated with CD3/CD28 beads on day 0. Following 24 and 48 h with activation, cells were subject to a first and second transduction with lentivirus encoding CAR19, CAR19/hIL12, and CAR19/hIL12ODD at variable multiplicity of infection (MOI = 1, 2, 5). 48 h after the second transduction, surface CD19-CARs expression on T cells was detected by fluorescence-activated cell sorting according to the percentage of positive cells (B) and mean fluorescence intensity (MFI) (C). Untransduced donor T (UTD) cells were used as control. (D) Western blot analysis of CD19-CAR expression. 17% = classic normoxia; 1% = artificially created hypoxia. Each data point represents triplicated technical replication, and each dataset represents mean ± SEM of triplicated independent samples.

The schematic representation of the lentiviral vector constructs used in this study is shown in Figure 1A. The second-generation CAR targeting human CD19 (CAR19) contained an anti-CD19 scFv domain derived from the FMC63 mouse hybridoma,^33^ along with the 4-1BB and CD3ζ signaling domains.^34^ The CAR19 construct was modified with a P2A element to co-express human IL-12 (hIL-12) that would be secreted continuously or an oxygen-sensitive hIL-12 that was fused with the oxygen-dependent degradation domain (ODD) of HIF1a^35^ at the C-terminal end. These are respectively designated CAR19/hIL12 and CAR19/hIL12ODD (Figure 1A). Lentiviral transduction of CD3/CD28-activated T cells from human healthy donors was carried out with variable multiplicity of infection (MOI = 1, 2, 5) for 48 h, and untransduced donor T (UTD) cells were used as control. CAR19 surface presentation was measured using flow cytometry according to the percentage of positive cells and mean fluorescence intensity (MFI). Donor T cells transduced with CAR19, CAR19/hIL12, or CAR19/hIL12ODD reached a plateau of ∼90% of CAR-positive cells at MOI = 5 (Figures 1B and S1), while the MFI continued to increase over the tested range of lentiviral particles doses (Figure 1C). Furthermore, CAR expression was also confirmed by Western blotting with anti-CD3ζAb after transduction at an MOI of 5 (Figure 1D). For subsequent work, the transduction of donor T cells was carried out using an MOI of 5.

### Hypoxia-regulated IL-12 secretion and cytolytic function of CAR19/hIL12ODD T cells *in vitro*

**Figure 2:**
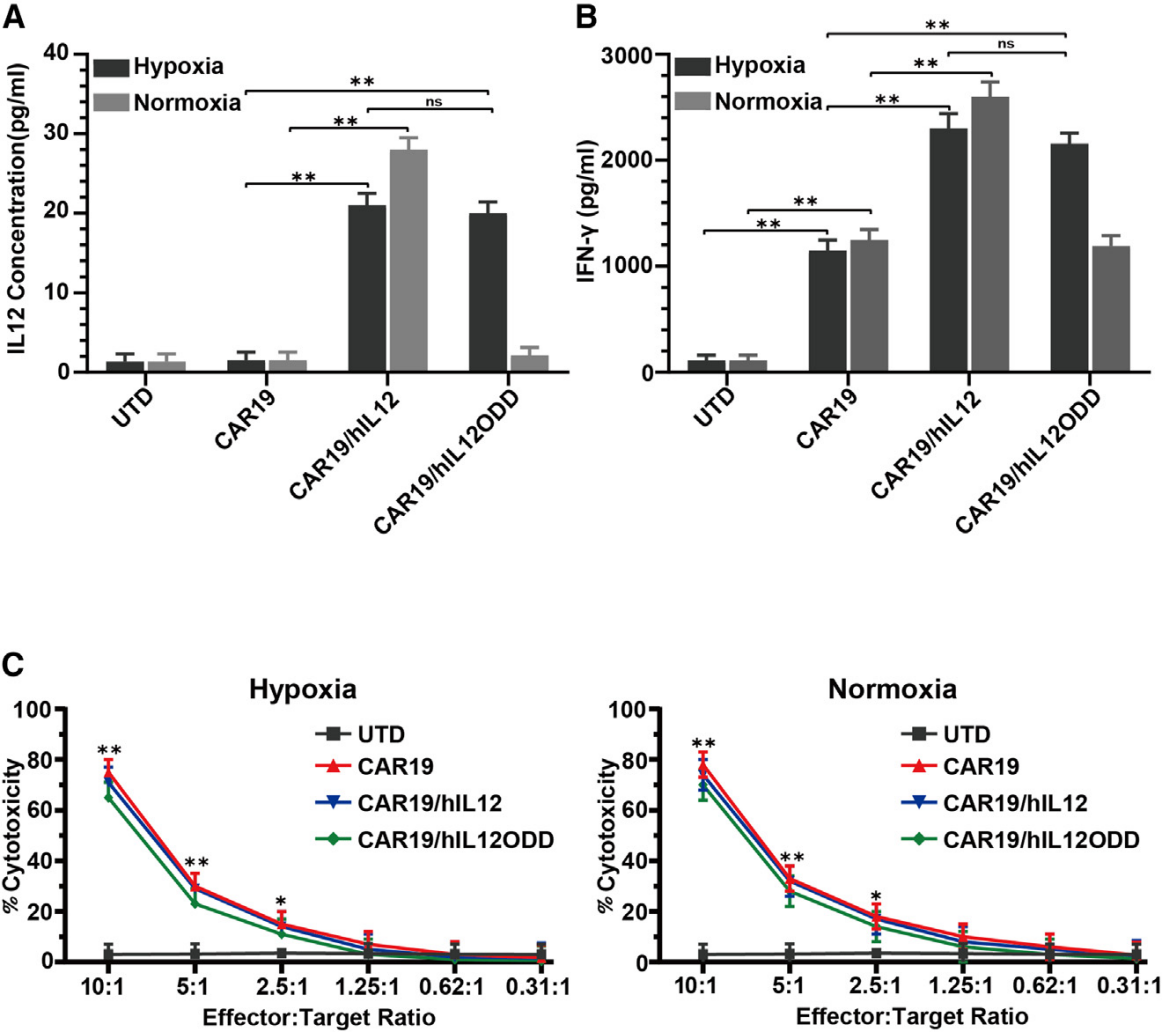
Hypoxia-dependent IL-12 production and *in vitro* cytolytic function of CAR19/hIL12ODD T cells (A and B) The secretion of bioactive IL-12 p70 and IFN-γ following 48-h culture or 72-h co-culture with OCI-Ly3 cells at a 1:1 ratio under hypoxic and normoxic environments. Data shown are the mean ± SEM of triplicates and compared using an independent t test, ∗∗p < 0.01. (C) *In vitro* cytotoxicity of T cells incubated with target cells at indicated ratios for 6 h under hypoxic and normoxic environments. Each dataset represents the mean ± SEM of one independent sample performed with triplicate wells.

To verify whether the IL-12 secretion capacity of CAR19/hIL12ODD T cells is dependent on the hypoxic environment, two oxygen concentrations, 17% for classic normoxia (based on the oxygen tension-dependent degradation of HIF1α in T cells^36^) and 1% for artificially created hypoxia, were chosen. Activated T cells transduced with different CARs (1 × 10^6^) and UTD cells were cultured for 48 h either in a hypoxic or normoxic environment. As expected, whether under the hypoxic or normoxic environments, CAR19/hIL12-T cells could release significant amounts of IL-12 into the culture supernatant compared to CAR19-T cells (20.67 ± 2.05 vs. 1.27 ± 0.21 pg/mL, p = 0.006; 26.13 ± 3.56 vs. 1.52 ± 0.24 pg/mL, p = 0.009) (Figure 2A). However, CAR19/hIL12ODD-T cells could only secrete IL-12 under hypoxic conditions, reaching amounts 15 times higher compared to CAR19-T cells (19.66 ± 1.24 vs. 1.27 ± 0.21 pg/mL, p = 0.003), which was nearly equivalent to IL-12 secretion by CAR19/hIL12-T cells in the same condition (19.66 ± 1.24 vs. 20.67 ± 2.05 pg/mL, p = 0.22) (Figure 2A). CAR19/hIL12ODD-T cells cultured in hypoxia were then transferred into normoxic conditions, and the decay of IL-12 in the culture supernatant was monitored over a 6-h period. IL-12 secretion from CAR19/hIL12ODD-T cells decreased by 80% in approximately 2 h (Figure S2A). These results demonstrated that CAR19/hIL12ODD T cells could produce significant amounts of IL-12, but only in a hypoxia-dependent manner, and that IL-12 secreted from CAR19/hIL12ODD T cells was degraded under normoxic conditions.

To explore the bioactivity of CAR-T cells expressing IL-12, we investigated expression and release of IFN-γ. The DLBCL cell line OCI-Ly3 with CD19 expression was co-cultured with various CAR-T or UTD cells at a ratio of 1:1 under hypoxia or normoxia for 72 h, and IFN-γ in the culture supernatant was detected using an ELISA. Apart from the UTD group, IFN-γ secretion was elevated in all groups (Figure 2B). Under both oxygen conditions, the amount of IFN-γ produced by CAR19/hIL12-T cells increased significantly in comparison with CAR19-T cells (2,306.3 ± 98.4 vs. 1,156.3 ± 59.7 pg/ml, p = 0.0005; 2,643.7 ± 86.9 vs. 1,262.7 ± 78.9 pg/ml, p = 0.0009) (Figure 2B). However, the CAR19/hIL12ODD-T cells produced a significantly increased amount of IFN-γ compared to CAR19-T cells only under hypoxic conditions (2,207.7 ± 123.5 vs. 1,156.3 ± 59.7 pg/ml, p = 0.0019) (Figure 2B). Of note, IFN-γ production by these CAR-T cells was very similar to IFN-γ production from CAR19/hIL12-T cells (2,207.7 ± 123.5 vs. 2,306.3 ± 98.4 pg/ml, p = 0.065) (Figure 2B). IL-2 production by these cells was also confirmed (Figure S2B). The cytolytic capacity of transduced T cells was determined by incubating individually various CAR-T or UTD cells with Ly3 cells. Lactate dehydrogenase (LDH) release assays demonstrated that both CAR19/hIL12ODD and CAR19/hIL12 could induce lysis under both oxygen conditions as effectively as CAR19 T cells (Figure 2C). In summary, these results confirmed that T cells transduced with CAR19/hIL12ODD could only secret bioactive IL-12 in a hypoxia-dependent manner, and they were able to lyse cognate tumor targets efficiently.

### Hypoxia-regulated IL-12 secretion results in rapid T cell expansion and a unique T cell phenotype

**Figure 3:**
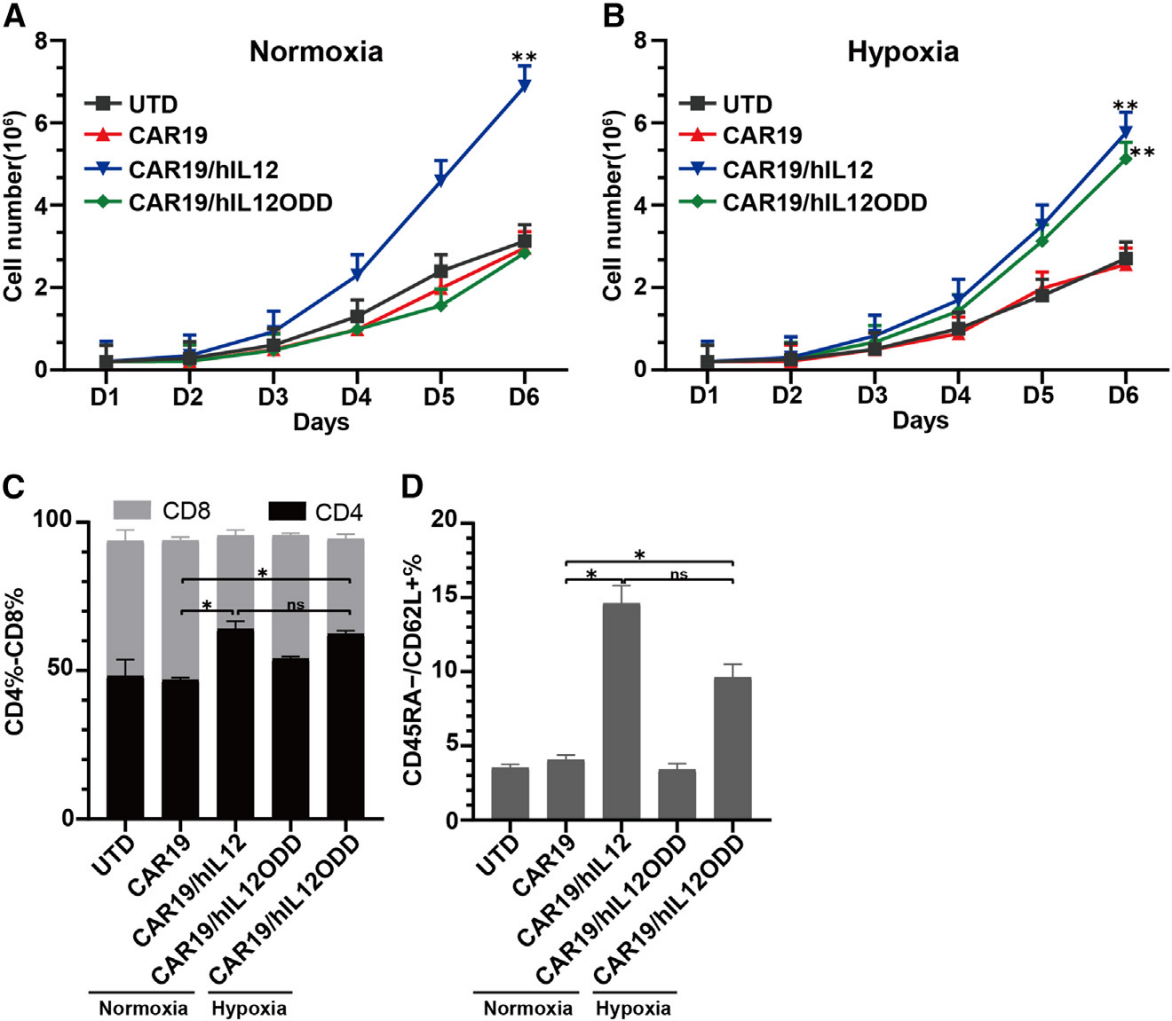
Secretion of IL-12 results in rapid expansion of T cells and an increased CD4, central memory T cell phenotype (A) Under normoxia, CAR19/hIL12-T cells with IL-12 secretion exhibited an elevated cell expansion over all other groups. (B) Under hypoxia, CAR19/hIL12ODD-T and CAR19/hIL12-T cells with IL-12 secretion displayed an elevated expansion efficiency compared with UTD and CAR19-T cell groups. Each dataset represents the mean ± SEM of triplicates and was compared using an independent t test. (n = 3, ∗∗p < 0.01). CAR19/hIL12-T (under normoxia) and CAR19/hIL12ODD-T (under hypoxia) cells in culture showed an increased CD4 ratio (C) and increased abundance of CD45RA^−^/CD62L^+^ central memory T cells (Tcm) (D). Each dataset is from three independent samples, and mean ± SEM is shown and was compared using an independent t test, ∗p < 0.05.

Human CD3^+^ T lymphocyte cells can be successfully isolated from human peripheral blood mononuclear cells (PBMCs) using CD3/CD28 beads. After lentiviral transfection, transduced CD3^+^ T cells must be induced to efficiently and rapidly proliferate to obtain sufficient quantity for clinical adoptive transfer therapy. By enumerating viable cells over 6 days, we demonstrated that IL-12 secretion can promote the expansion of human CD3^+^ T lymphocyte cells. Under normoxic conditions, only CAR19/hIL12-T cells showed a significantly more rapid proliferation rate compared to other groups (Figure 3A) accompanied by detectable levels of IL-12 in the cell culture supernatant. However, under hypoxic conditions, CAR19/hIL12ODD-T cells exhibited almost the same proliferation rate (Figure 3B) as CAR19/hIL12-T cells (5.15 ± 0.12 × 10^6^ vs. 5.69 ± 0.13 × 10^6^, p = 0.09), which were significantly higher compared to UTD (5.15 ± 0.12 × 10^6^ vs. 2.76 ± 0.24 × 10^6^, p = 0.001) and CAR19-T (5.15 ± 0.12 × 10^6^ vs. 2.58 ± 0.14 × 10^6^, p = 0.001) cell groups. Flow cytometry was used to determine the ratio of CD4:CD8 and the expression of memory phenotype markers CD45RA and CD62L on these cells. T cells expanded in the context of IL-12 were enriched for CD4^+^ (Figures 3C and S3) and showed an increased abundance of CD45RA^−^/CD62L^+^ central memory T cells (Tcm) (Figures 3D and S3). From these results, we discovered that exogenous secretion of IL-12 can support rapid expansion of T cells and an increase in CD4 and central memory T cell phenotypes, ideal for clinical adoptive transfer therapy.

### Improved antitumor activity of IL-12-secreting CD19 CAR

**Figure 4:**
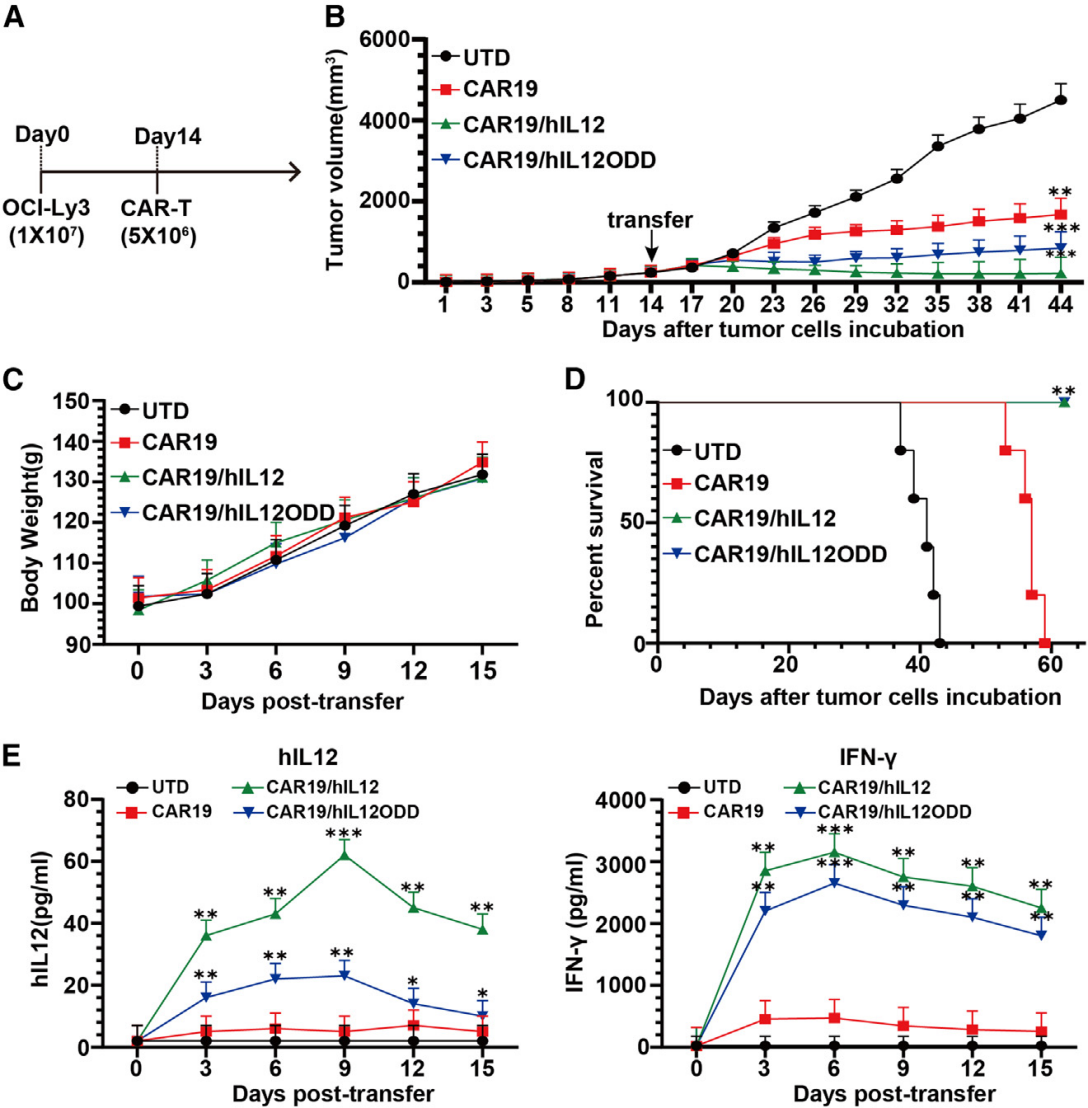
Improved antitumor activity of IL-12 secreting CD19 CAR-T cells A total of 1 × 10^7^ OCI-Ly3 cells were inoculated into the right flank of 6-week-old Syrian hamsters with an immune-deficient phenotype (named ZZU001) on day 0. (A) Experimental scheme of the *in vivo* therapeutic efficacy experiment. On day 14, five ZZU001 mice per group bearing tumors of 250∼300mm^3^ were infused intravenously with 5 × 10^6^ T cells as indicated. (B) Growth curve of OCI-Ly3 xenografts treated with the indicated T cells. The arrow indicates T cell infusion. Mean tumor size and SEM are shown for each group and compared using a one-way ANOVA with post hoc Tukey’s Multiple Comparison Test, ∗∗p < 0.01. (C) Body weights of each group were measured on days 3, 6, 9, 12, and 15 post transfer. Mean and SEM are shown. (D) Kaplan-Meier survival curves were generated, and a log rank (Mantel-Cox) test was used to analyze significance, ∗∗p < 0.01. (E) The amount of IL-12 and IFN-γ in sera on days 3, 6, 9, 12, 15 post transfer was detected by ELISA. Mean and SEM are shown. Statistical analysis was carried out using an independent t test and compared to the CAR19 group, ∗∗p < 0.01, ∗∗∗p < 0.001.

To evaluate the *in vivo* therapeutic efficacy of CAR-T cells, the subcutaneous OCI-Ly3 xenograft tumor model was established in Syrian hamsters with an immune-deficient phenotype caused by knockout of interleukin-2 receptor subunit gamma (*IL2RG*) (named ZZU001).^32^ The therapeutic dosage for CAR-T cells was determined by performing the human-to-hamster dose conversion using the equation as described previously.^37^ T cells engineered with CAR19, CAR19/hIL12, CAR19/hIL12ODD, or UTD T cells cultured under normoxic conditions over 1 week (5 × 10^6^ positive for each group) were adoptively transferred intravenously into ZZU001 mice when tumors reached 250∼300 mm^3^, a size at which a hypoxic environment is predicted. (Figures 4A and S4A). Analysis of the growth of subcutaneous OCI-Ly3 tumors demonstrated that both IL-12-secreting CD19 CARs (CAR19/hIL12 and CAR19/hIL12ODD) prevented tumor outgrowth by 30 days compared to CAR19 and UTD T cell groups (Figures 4B and S4B). As a proxy for overall animal health, body weight was monitored, and no significant change was observed between the treatment groups (Figure 4C). Treatment with CAR19/hIL12 and CAR19/hIL12ODD T cells significantly improved survival compared with CAR19- and UTD-treated animals (Figure 4D). To elucidate the enhanced antitumor mechanism associated with CAR19/hIL12 and CAR19/hIL12ODD T cells, the changes in amount of IL-12(p70) and IFN-γ in the sera of ZZU001 mice were monitored at various time points (days 3, 6, 9 12, 15) following the transfer of CAR-T cells. As shown in Figure 4E, compared to CAR19-T cells, significant systemic IL-12 levels were detected in sera from animals treated with CAR19/hIL12-T cells, but not with CAR19/hIL12ODD-T cells, throughout the time points analyzed, which peaked at 60 pg/mL on day 9 of post transfer (61.45 ± 4.62 vs. 6.21 ± 0.78 pg/mL, p < 0.001; 18.32 ± 2.01 vs. 6.21 ± 0.78 pg/mL, p = 0.052). Real-time PCR detection of DNA encoding anti-CD19 CAR from the animal’s residual whole blood cell showed that, compared to CAR19-T cells, there was a peak level of more than a 2-log and nearly 1.5-log expansion for CAR19/hIL12-T and CAR19/hIL12ODD-T cells (Figure S4C). Both CAR19/hIL12 and CAR19/hIL12ODD-T cells caused increased IFN-γ levels, and these were raised to a significantly greater degree than after treatment with CAR19-T cells (2,750.62 ± 109.21 vs. 345.54 ± 22.54 pg/mL, p < 0.001; 2,295.41 ± 86.33 vs. 345.54 ± 22.54 pg/mL, p < 0.001) (Figure 4E). These results indicated that expression of IL-12 by the CD19 CAR-T system can significantly improve the antitumor effect and that regulating IL-12 expression using ODD reduces the amount of systemic IL-12 detected after treatment compared to an unregulated IL-12 expression system.

### CAR19/hIL12ODD T cells can safely cure OCI-Ly3 xenografts model

**Figure 5:**
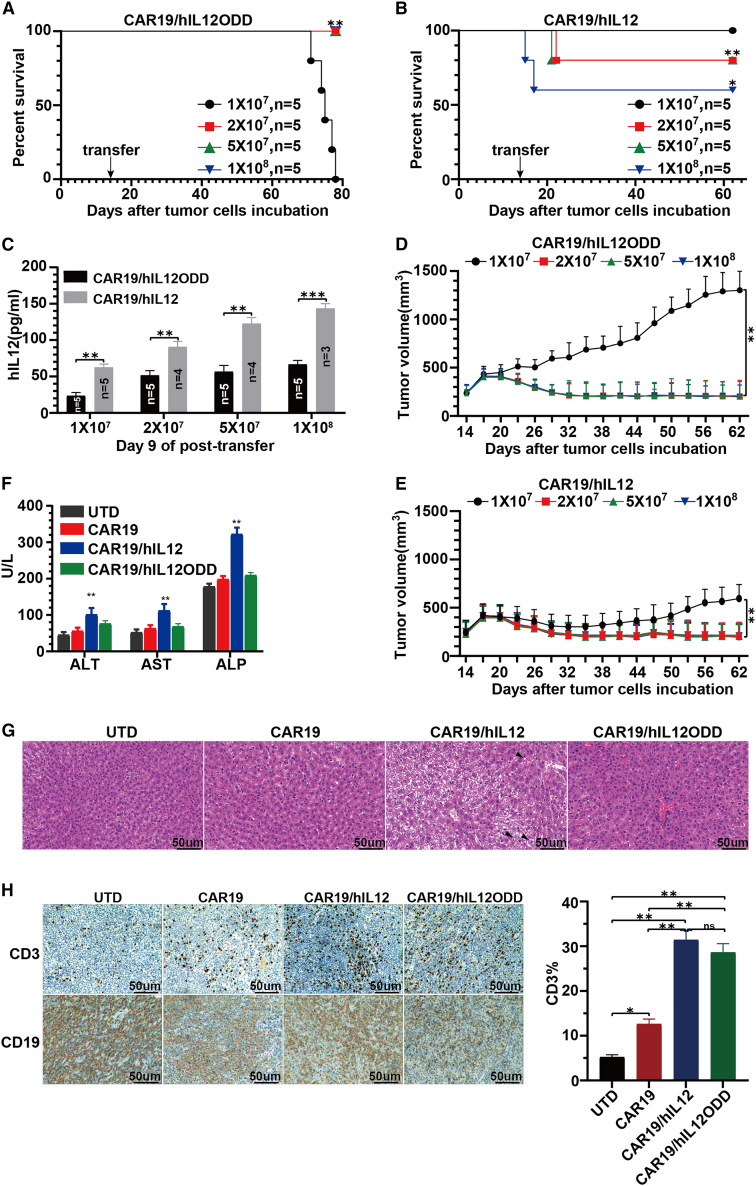
CAR19/hIL12ODD is a safe and effective treatment for subcutaneous OCI-Ly3 xenograft tumors in hamsters 14 days after 1 × 10^7^ OCI-Ly3 cells were seeded on the right flank of an immune-deficient Syrian hamster model (named ZZU001), five ZZU001 per group bearing tumors of 250∼300mm^3^ were infused intravenously with indicated doses (1 × 10^7^, 2 × 10^7^, 5 × 10^7^, and 1 × 10^8^) of CAR19/hIL12ODD (A) or CAR19/hIL12 T cells (B). Survival was monitored and displayed using Kaplan-Meier survival curves. Significance was assessed using the log rank (Mantel-Cox) test, ∗p < 0.05, ∗∗p < 0.01. (C) IL-12 in sera on day 9 post transfer was detected by ELISA. Mean and SEM are shown. Statistical analysis was carried out using an independent t test, ∗∗p < 0.01, ∗∗∗p < 0.001. Mean tumor size and SEM are shown for CAR19/hIL12ODD (D) and CAR19/hIL12 (E) T cell groups in the dose escalation study and compared using a one-way ANOVA with post hoc Tukey’s Multiple Comparison Test, ∗∗p < 0.01. Using the same model, three ZZU001 per group were injected intravenously with CAR19/hIL12ODD, CAR19/hIL12, CAR19, or UTD T cells at a dose of 2 × 10^7^. (F) Serum was collected on day 3 for detection of the level of ALT, AST, and ALP. Mean and SEM are shown. Statistical analysis was carried out using an independent t test, ∗∗p < 0.01. (G) Representative histopathology of the liver after injection of indicated T cells at a dose of 2 × 10^7^. Livers were collected on day 3 following infusion and analyzed using H&E staining (200×). (H) Representative images of immunohistochemical staining for CD3 and CD19 within the tumor at day 3. CD3-positive cells were counted in five high-power fields from each tumor section (200×). Each dataset represents mean ± SEM and significance was assessed using an independent t test, ∗p < 0.05, ∗∗p < 0.01.

IL-12-secreting CD19 CAR-T (CAR19/hIL12, CAR19/hIL12ODD) cells exhibit a significantly enhanced antitumor efficacy. However, as an immunostimulatory cytokine, application of IL-12 is limited clinically by its potential for systemic toxicity. Our previous report demonstrated that the Syrian hamster is an effective model for assessing hIL-12-induced inflammatory cytokine syndrome and systemic toxicity that is manifested as hepatic dysfunction.^31^ To assess the improvement in safety associated with CAR19/hIL12ODD T cells over CAR19/hIL12 T cells, a dose escalation study was conducted. The OCI-Ly3 xenograft-bearing ZZU001 mice were treated intravenously using increasing doses (1 × 10^7^, 2 × 10^7^, 5 × 10^7^, and 1 × 10^8^) of CAR19/hIL12 or CAR19/hIL12ODD T cells. As shown in Figures 5A and S5A, CAR19/hIL12ODD treatment with the dosage of 2 × 10^7^, 5 × 10^7^, and 1 × 10^8^ resulted in 100% survival, which persisted until termination of the experiment. No animal exhibited signs of treatment-related toxicity. In contrast, each dose of CAR19/hIL12, except for the dosage of 1 × 10^7^, led to various degrees of death (20%–40%) within a week following infusion. In particular, the dosage of 1 × 10^8^ resulted in 40% death within 4 days of infusion (Figures 5B and S5A). As expected, compared to CAR19/hIL12ODD, serum IL-12 levels were significantly higher after treatment with CAR19/hIL12 throughout the time points analyzed, peaking at day 9 (143.23 ± 7.54 vs. 61.52 ± 3.63 pg/mL, p < 0.001) (Figures 5C and S5B). Following the dose escalation regimen, analysis of the growth of subcutaneous OCI-Ly3 tumors demonstrated that the dose of 2 × 10^7^ was optimal for transfer as animals receiving 2 × 10^7^ or higher doses of CAR19/hIL12ODD showed a consistent response to treatment (effectively cured) (Figure 5D). Transfer of 2 × 10^7^ CAR19/hIL12 cells also achieved an identical efficacy response (Figure 5E), but with the early post-infusion death of some animals (Figure 5B).

To further confirm the improvement in safety and the capacity for remodeling the tumor immune microenvironment, a safety confirmation study was conducted. The OCI-Ly3 xenograft-bearing ZZU001 were treated intravenously with CAR19/hIL12ODD, CAR19/hIL12, CAR19, or UTD T cells at a dose of 2 × 10^7^ cells. Liver toxicity was assessed by measuring alanine aminotransferase (ALT), aspartate aminotransferase (AST), and alkaline phosphatase (ALP) levels in the serum on day 3 following injection. Significant elevations in all three enzymes were detected in the CAR19/hIL12 T cell-treated group, whereas liver enzymes measured in CAR19/hIL12ODD T cell-treated animals remained equivalent to those detected in CAR19 or UTD T cell groups (Figure 5F). Moreover, pathological examination of livers after treatment with CAR19/hIL12ODD and CAR19 T cells demonstrated only mild blood vessel congestion, while CAR19/hIL12 induced severe blood vessel congestion, apoptosis, and necrosis of hepatocytes (Figure 5G). In addition, immunohistochemical staining for CD3 showed enhanced CD3^+^ T cell infiltration within tumor tissue after treatment with CAR19/hIL12ODD and CAR19/hIL12 T cells compared to treatment with CAR19 T cells (Figure 5H). In summary, these results demonstrated that CAR19/hIL12ODD T cells can safely cure the subcutaneous DLBCL xenograft model.

## Discussion

CD19 is an attractive therapeutic target since it is expressed in over 95% of B cell malignancies. CD19 CAR-T cell therapy has been shown to be an attractive therapeutic strategy for relapsed/refractory B cell malignancies. Several clinical research centers have adopted the CD19 CAR-T cells for relapsed/refractory B-ALL, and up to 81%–93% of patients achieved CR within the follow-up time.^9^^,^^10^^,^^38^ However, the phase II clinical trial conducted by Kite/Gilead^39^^,^^40^ (NCT02348216) and JULIET^41^ (NCT02445248) for treating relapsed/refractory DLBCL only achieved a CR rate of 58% in long-term follow-up data. The clinical success in B-ALL has failed to translate into DLBCL to a large extent because of physical barriers to therapy and the immunosuppressive tumor microenvironment.^12^^,^^13^ These obstacles limit the proliferation and persistence of CAR-T cells and impair the antitumor efficiency of CD19 CAR-T cells. Many approaches have been implemented in an attempt to improve the efficacy of CAR-T cells via overcoming the suppressive effects of microenvironment.^42^ Genetic modifications to CAR constructs with pro-inflammatory cytokines or ligand have also been reported.^43^^,^^44^

Given its potential to mediate tumor regression in xenograft mouse models for a variety of cancers,^45^ IL-12 has been clinically applied to cancer patients since 1994.^22^ However, potentially lethal toxicity associated with systemic administration precludes its clinical adoption.^46^^,^^47^ In order to control the toxicity of IL-12, researchers have attempted to develop new delivery systems to localize IL-12 at tumor sites. In 2004, Wagner and colleagues first reported that IL-12 could be locally delivered to tumor sites of Epstein-Barr virus (EBV)-positive Hodgkin’s disease by EBV-specific cytotoxic T lymphocytes (CTLs).^48^ IL-12-transduced EBV-specific CTLs demonstrated a functional advantage for overcoming the adverse tumor immunosuppressive environment. However, the complicated procedure to obtain tumor-specific T cells impeded its general application. Rosenberg and colleagues have genetically engineered patients’ T cells with a retroviral vector containing an inducible single-chain IL-12 gene driven by an NFAT-responsive promoter (NFAT.IL12), which can induce the secretion of IL-12 following specific antigen recognition via the T cell receptor.^27^^,^^49^^,^^50^ NFAT.IL12-transduced T cells demonstrated a significantly enhanced therapeutic effect, notably without toxicity in murine tumor model.^50^ However, in the human trial, NFAT.IL12 treatment was associated with high serum levels of IL-12 as well as clinical toxicities including hepatic dysfunction and high fevers.^27^

In our work, CD3 T cells derived from human PBMCs were transduced with a lentiviral vector in which CD19 CAR was combined with hypoxia-dependent IL-12 through P2A peptides by fusion of IL-12 to the ODD domain of HIF1α. This approach was undertaken in view of the lower oxygen tension accompanied with HIF1α^28^^,^^29^ expression in lymph nodes where DLBCL usually occurs, in contrast to the blood that exhibits a normoxic environment.^51^ Cleaved CAR19 expression and surface presentation were confirmed (Figures 1C and 1D). In theory, under normoxic conditions, ODD results in IL-12 degradation. Secretion of bioactive IL-12 occurs only under hypoxic conditions. This refinement only permits those CD19 CAR-T cells that traffic to the tumor site to secrete IL-12 once the hypoxic environment in the tumor is encountered. Previous reports have demonstrated significantly enhanced tumor regression or eradication associated with IL-12-secreting CAR-T cells in xenograft mouse models for a variety of cancers, including melanoma,^50^ hepatocellular carcinoma,^26^ and ovarian cancer.^15^ However, all of these studies were conducted on relatively small tumors (≦50 mm^3^) size. In contrast, our novel strategy using regulated IL-12-secreting CAR-T cells has demonstrated an improvement in antitumor activity for treatment of large tumors (∼250 mm^3^). In addition, it has been demonstrated that the OCI-ly3 cell line is a model that reflects a clinical relapsed/refractory scenario.^52^^,^^53^ Therefore, the therapeutic effect associated with our strategy is more reflective of the clinical situation.

The preclinical assessment of toxicity associated with human IL-12-secreting CAR-T cells plays a very important role in its clinical application. It had been reported that human IL-12 cannot stimulate mouse PBMCs.^54^ Our previous report confirms this, but we have demonstrated that human IL-12 can stimulate both human and hamster PBMC proliferation. Moreover, human IL-12 is capable of stimulating the expression of IFN-γ and TNF-α by activated splenocytes *ex vivo* in hamster models. Therefore, the Syrian hamster is an effective model for assessing IL-12-induced inflammatory cytokine syndrome and systemic toxicity in humans.^31^ Indeed, using the Syrian hamster model generated with a severe immune deficiency in T and B cells (ZZU001),^32^ toxicity was observed after administration of >2 × 10^7^ CAR19/hIL12 T cells that produce IL-12 systemically, which is manifested as hepatic dysfunction (Figures 5C and 5G). However, when adoptively transferring up to 1 × 10^8^ CAR19/hIL12ODD T cells, we did not observe any toxicity. Further, kidney injury was not observed in the treatment of safety confirmation study (Figure S5C). Analysis of IL-12 in the serum suggested that 60 pg/ml of IL-12 in serum could be tolerated. This was also consistent with our previous study for an orthotopic pancreatic cancer (PaCa) model treated with oncolytic adenovirus (Ad-TD-nsIL-12) harboring a modified IL-12.^31^

The mechanism for antitumor activity with IL-12 is under investigation. One possible explanation is that it can boost the production of IFN-γ, which is the most potent mediator of IL-12 actions.^55^ In our work, the CAR19/hIL12ODD T cells demonstrated excellent therapeutic efficacy accompanied with increased IFN-γ in animal models. However, while IFN-γ levels were raised after treatment, no changes in response to dose escalation were noted. On the contrary, CAR19/hIL12 T cells exhibited significantly increased IFN-γ at the analyzed time points as the input dose was increased (Figure S5D). Measurement of serum IL-12 levels confirmed that ODD can efficiently regulate IL-12 *in vivo*. Another explanation is that tumor antigen-specific T cells deliver IL-12 to the tumor microenvironment where it can enhance the endogenous immune system to infiltrate and destroy tumors. Agliardi et al. recently reported that an intratumoral IL-12 delivery combined with CAR-T cells targeting tumor-specific epidermal growth factor receptor variant III (EGFRvIII) can drive increased infiltration of T cells in an orthotopic glioblastoma multiforme mouse model.^56^ The CAR19/hIL12ODD T cells showed excellent therapeutic efficacy in our animal models and suggested enhanced T cell infiltration within tumor tissue. However, given the stimulative effect of human IL-12 on hamster PBMCs and possible immune deficiency in natural killer cells in the ZZU001 model, a role for natural killer cells cannot be ruled out.

In summary, our research has demonstrated that hypoxia-dependent synthesis of IL-12 could boost the function of CD19 CAR-T cells with fewer side effects compared to use of unmodified IL-12. These findings could broaden the application of CAR-T cells-based immunotherapy to patients suffering from relapsed or refractory DLBCL and might be an alternative therapeutic strategy for patients with certain types of solid tumors.

## Materials and methods

### Cell lines

Human DLBCL cell line OCI-Ly3 and human embryonic kidney (HEK)-293T cell line were cultured as previously described.^52^^,^^53^

### Isolation and expansion of T cells

Human PBMCs derived from healthy human donors were obtained from the Affiliated Cancer Hospital of Zhengzhou University & Henan Cancer Hospital (License 2019341). Human CD3^+^ T cells were isolated, activated, and expanded from PBMCs using Dynabeads CD3/CD28 CTS (Gibco) according to the manufacturer’s instructions. The derived T cells were cultured as previously described.^26^

### CAR expression vector construction

All lentivirus vector constructs used in this study are schematically illustrated in Figure 1A. A second-generation CAR targeting human CD19, named CAR19, uses the anti-CD19 single-chain variable fragment (scFv) linked in-frame to the human CD8a hinge and transmembrane region, the human 4-1BB intracellular signaling domain, and CD3ζ signaling domain, which was synthesized by GeneArt Gene Synthesis (Invitrogen). CAR19/hIL12 was generated by fusing CAR19 with human IL-12 p70 (IL12p40-GTTCCTGGAGTAGGGGTACCTGGGGTGGGC-IL12p35) using the porcine teschovirus-1 2A (P2A) peptide. The ODD of HIF1α was fused in-frame to the C-terminal end of hIL-12 p70 to generate CAR19/hIL12ODD.

### Flow cytometry assays

CAR19 expression was detected by staining T cells at 48 h after transduction using a fusion protein containing human CD19 extra-cellular fragment (AA 1–291) linked in-frame with mouse IgG Fc portion and then followed by F(ab')2-Goat anti-Mouse IgG (H + L) Secondary Antibody (eBioscience). The mouse IgG Fc portion protein was used as an isotype control. Expanded T cells were analyzed by flow cytometry after staining with the following antibodies according to the manufacturer’s instructions: PE-conjugated antibodies specific for human CD4 (OKT4), APC-conjugated CD8 (HIT8a), FITC-conjugated CD45RA (HI100), and APC-conjugated CD62L (DREG-56) obtained from eBioscience.

### Cytotoxicity assays

OCI-Ly3 cells were co-cultured with the CAR-T cells at gradient effector/target ratio. Following 6 h of co-culture, the levels of supernatant LDH were tested using the CytoTox 96 Non-Radioactive Cytotoxicity Kit (Promega) in accordance with the manufacturer’s instructions. All assays were performed at least three times.

### Cytokine release assays

The levels of hIL-12 p70 and IFN-γ-secreted in cell culture supernatant were detected by using a commercial IL-12 p70 Human ELISA Kit (Invitrogen) and IFN-γ Human ELISA Kit (Invitrogen) according to the manufacturer’s manual. For these assays, 5 × 10^5^ transduced cells were incubated (48 h for IL-12 p70) or co-incubated with 5 × 10^5^ target cells (72 h for IFN-γ) under two oxygen concentrations (17% for classic normoxia and 1% for artificially created hypoxia) in 1 mL of culture volume in individual wells of 24-well plates respectively. Cytokine secretion was measured in culture supernatant diluted to the linear range of the assay. For the serum cytokine release assay, ZZU001 sera were collected by centrifuging peripheral blood at 1,000 *g* for 30 min and then measured by ELISA kits. All the samples were analyzed in triplicate.

### Xenograft tumor models

For the established OCI-Ly3 xenograft tumor models, 6-week-old ZZU001 hamsters^32^ were inoculated subcutaneously with 1 × 10^7^ OCI-Ly3 cells on the right flank. When the tumor lesions were ∼250–300 mm^3^, animals were randomly divided into indicated groups (n = 5) and subsequently injected intravenously with the indicated CAR-T cells. Tumor size was measured every 3 days with calipers, and the volume was calculated using the formula: (Π × Length × Width^2^)/6. Animals were killed when the tumor size reached 3,500 mm^3^. All animals were housed and treated in accordance with the Provision and General Recommendation of Chinese Experimental Animals Administration Legislation. The study was reviewed and approved by the Ethical Committee of Zhengzhou University (License ZZU-LAC20210625[11]).

### Histopathological examination and immunohistochemistry

The tissues collected at the indicated time points were processed and stained by H&E staining or immunohistochemistry (IHC) for CD3 (eBioscience, cat. no. 14-0038-82) as previously described.^31^^,^^57^

### Statistical analysis

Statistical analysis was carried out using Graph Pad Prism 8 and SPSS 19.0 software. For quantitative variables, Kolmogorov-Smirnov test was used to test the normality of each group’s distribution. After confirmation of the normally distribution of each variable, the results were represented as mean ± standard deviation or ± standard error of the mean (SEM). Differences between groups were analyzed using the Student’s t test, one-way ANOVA test, or Kaplan-Meier survival analysis. Differences were considered statistically significant when the p value was less than 0.05.

## Data and code availability

For original data, please contact yaohe.wang@qmul.ac.uk.

## Supplemental information

Document S1. Figures S1–S5

Document S2. Article plus supplemental information
