# Supplementary material for: Hypoxia-regulated secretion of IL-12 enhances antitumor activity and safety of CD19 CAR-T cells in the treatment of DLBCL
Source: Mol Ther Oncolytics. 2023 Aug 18;30:216–26. doi: 10.1016/j.omto.2023.08.009 (PMC10471514; doi:10.1016/j.omto.2023.08.009)
Supplement: Document S1. Figures S1–S5 [file mmc1.pdf]

OMTO, Volume *30*

Supplemental information

Hypoxia-regulated secretion of IL-12 enhances antitumor activity and safety of CD19 CAR-T cells in the treatment of DLBCL

Wenping Zhou, Jinxin Miao, Zhenguo Cheng, Zhimin Wang, Jianyao Wang, Haoran Guo, Pengju Wang, Shuangshuang Lu, Lingling Si, Zhongxian Zhang, Louisa Chard Dunmall, Yanyan Liu, Nicholas R. Lemoine, and Yaohe Wang

Figure S1

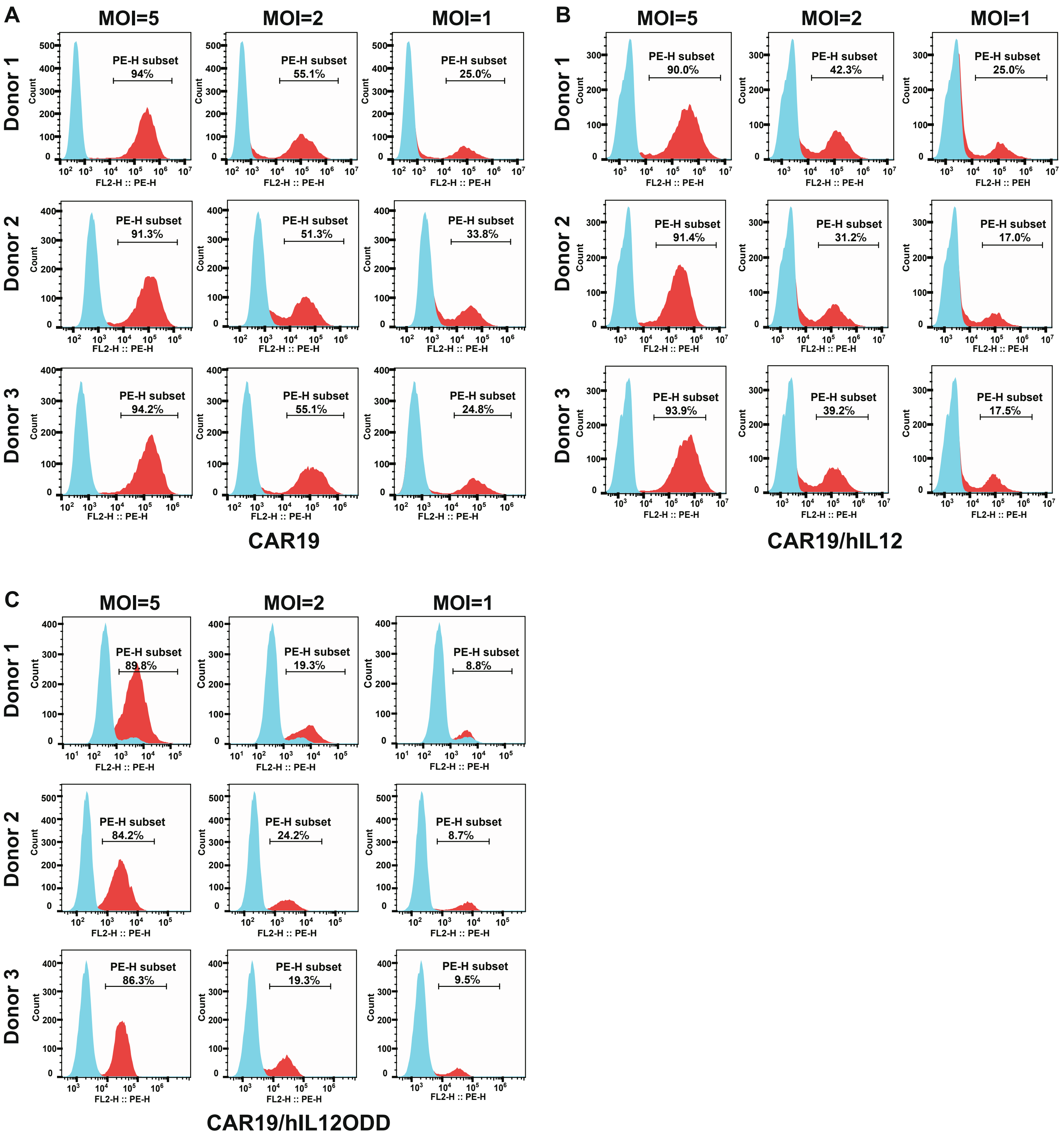

Figure S1: Flow cytometric analysis of transduction efficiency for three sets of donor T cells after 48 hours of transduction with lentivirus encoding CAR19 (A), CAR19/hIL12 (B) and CAR19/hIL12ODD (C) at variable multiplicities of infection (MOI=1, 2, 5), Untransduced donor T (UTD) cells were used as control.

Figure S2

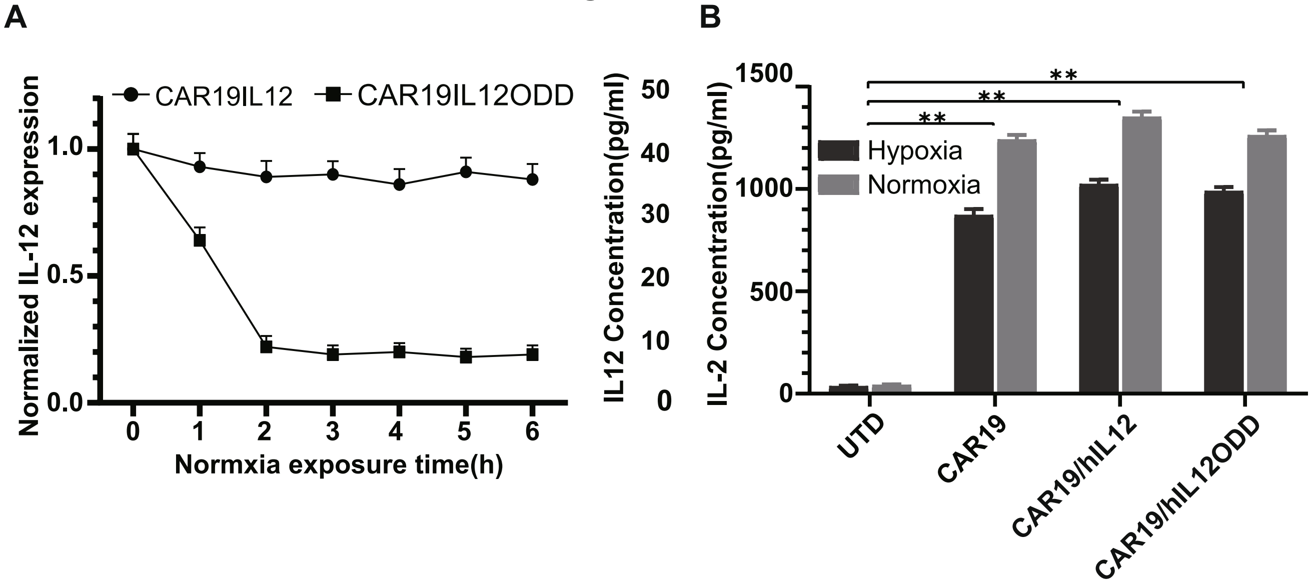

Figure S2: (A) Time course analysis of IL-12 secretion decay after removal of the hypoxia input, the secretion amounts were normalized to 1 for quantity at the time of normoxia reestablished. (B) The secretion of IL-2p following 72 hours co-culture with OCI-Ly3 cells at a 1:1 ratio under hypoxic and normoxic environments.

Figure S3

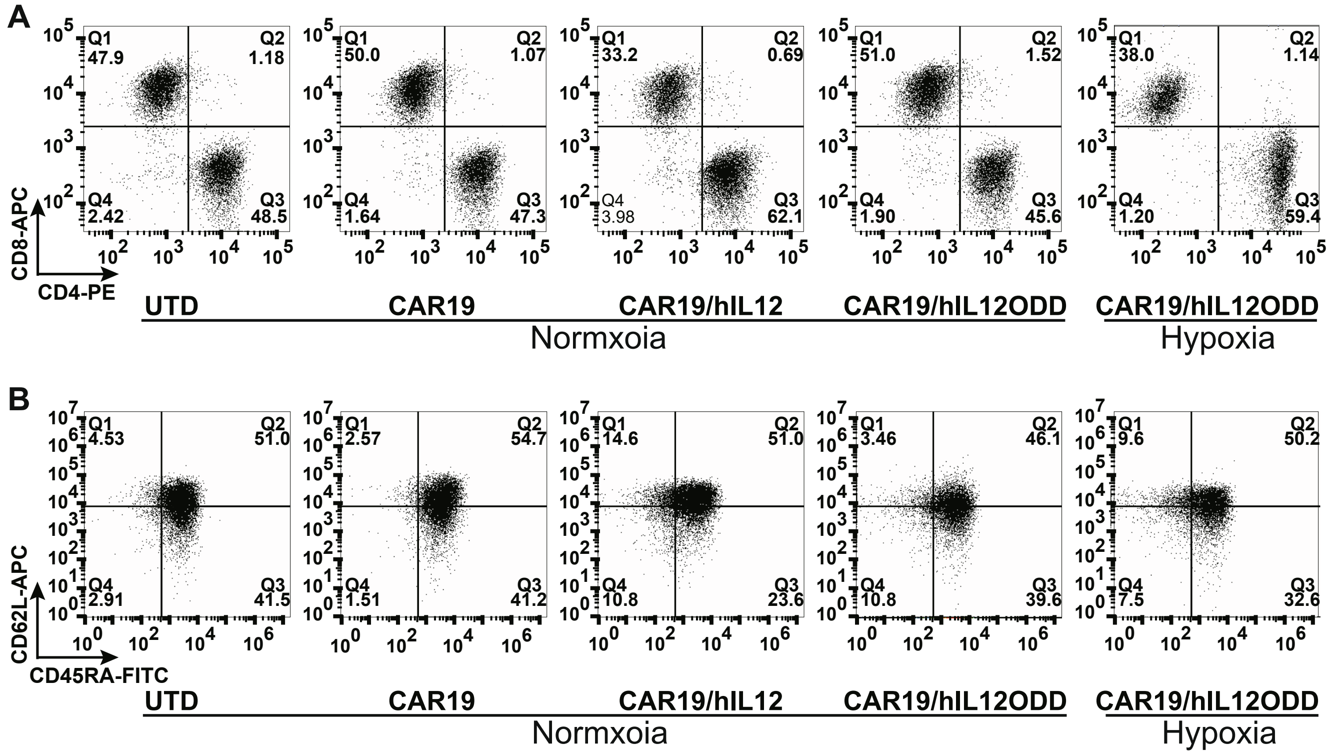

Figure S3: Flow cytometric analysis showed, CAR19/hIL12-T (under normxoia) and CAR19/hIL12ODD-T (under hypoxia) cells cultured with the secretion of IL-12 showed an increased CD4 ratio (A) and increased abundance of CD45RA−/CD62L+ central memory T cells (Tcm) (B). Data shown are representative of one independent sample.

Figure S4

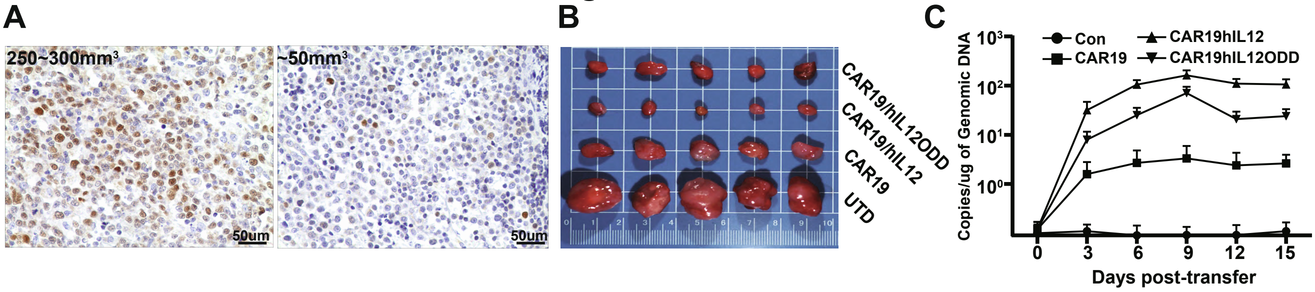

Figure S4: (A) Representative images of IHC staining for HIF1α within the tumor that exhibited hypoxia when reached 250~300mm³. (B) IL-12-secreting CD19 CARs (CAR19/hIL12 and CAR19/hIL12ODD) caused the prevented tumors outgrowth by 30 days compared to CAR19 and UTD T cell groups. (C) Real-time PCR detected DNA encoding anti-CD19 CAR showed that, compared to CAR19-T cells, a peak level of more than a 2-log and nearly 1.5-log expansion for CAR19/hIL12-T and CAR19/hIL12ODD-T cells in vivo by day 9 after infusion.

Figure S5

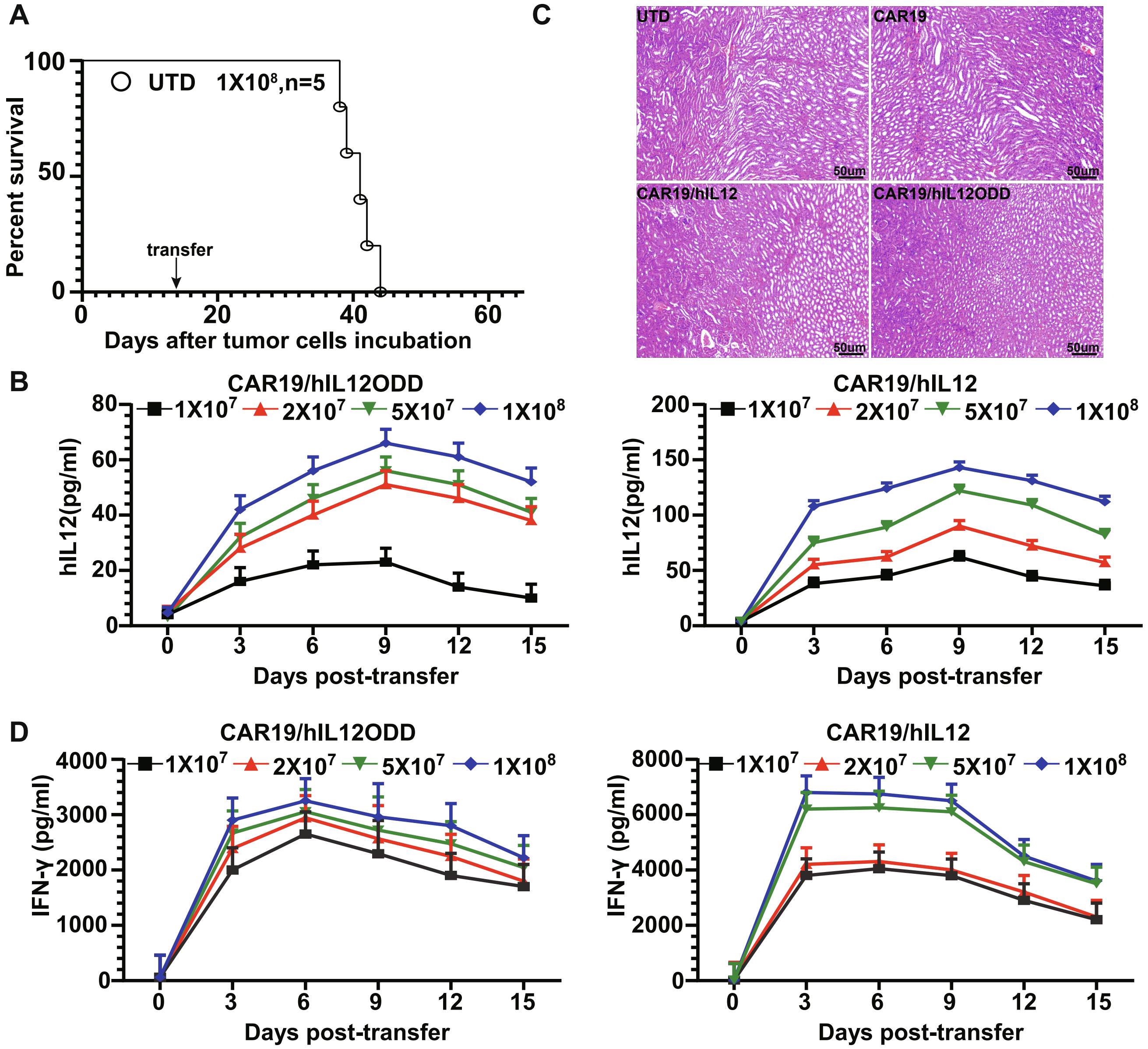

Figure S5: (A) The survival curve for OCI-Ly3 xenograft-bearing ZZU001 infused with the highest dosage of UTD cells (1×10⁸) that was set up as the control for CAR19/hIL12ODD and CAR19/hIL12 T cells in the dose escalation study.The amount of IL-12 (B) and IFN-γ (D) in sera on day 0, 3, 6, 9, 12, 15 post transfer was detected by ELISA in the dose escalation study. Mean and SEM are shown. (C) Representative histopathology of the kidney in the treatment of safety confirmation study.
